# Looking towards the future of MRI in Africa

**DOI:** 10.1038/s41467-024-46567-3

**Published:** 2024-03-13

## Abstract

Magnetic Resonance Imaging (MRI) is a crucial diagnostic tool within modern healthcare, yet its availability remains largely confined to high-income nations. The imperative to extend MRI accessibility to lower-income countries aligns with the pursuit of universal health coverage, a key target of the UN’s Sustainable Development Goal 3. In an interview with *Nature Communications*, three scientists dedicated to advancing MRI accessibility in Africa share their insights. These experts include *Dr Udunna Anazodo* (Assistant Professor at McGill University, Canada and Scientific Director, Medical Artificial Intelligence (MAI) Lab, Lagos, Nigeria), *Dr Johnes Obungoloch* (Lecturer at Mbarara University of Science and Technology, Uganda) and *Dr Ugumba Kwikima* (Neuroradiologist, Lugalo General Military Hospital, Tanzania). Our discussion considers the current MRI landscape across African countries and the associated challenges and opportunities. We also cover technological innovations making a difference, such as low field MRI, alongside the role of advocacy initiatives in bolstering accessibility. We finally look ahead to the future of MRI in Africa.

1. What is your research background and how do you know each other?

*Ugumba Kwikima*: I am a neuroradiologist based in Dar es Salaam, Tanzania with an interest in AI and data science. I got to know Udunna when participating in the Sprint AI Training for African Medical Imaging Knowledge Translation (SPARK) Academy [https://www.cameramriafrica.org/spark], a training program to prepare African imaging researchers like myself to participate in the Brain Tumour Segmentation Challenge Africa (BraTS-Africa)^1^ held at the Medical Image Computing and Computer Assisted Intervention (MICCAI) 2023 conference. I met Johnes during the first International Society of Magnetic Resonance in Medicine (ISMRM) African chapter in Ghana, which took place in September 2023.

**Figure:**
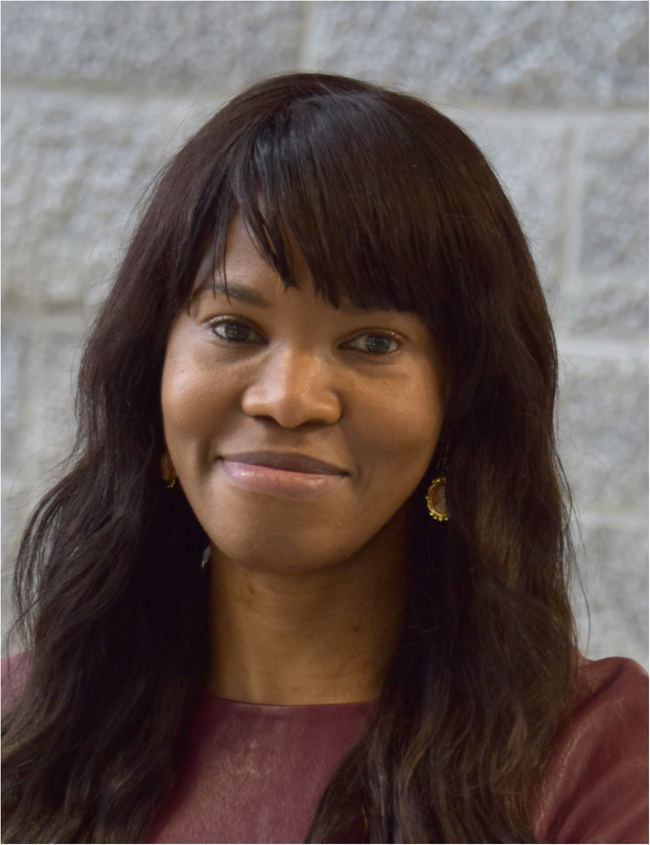
Udunna Anazodo, Assistant Professor at McGill University, Canada and Scientific Director, Medical Artificial Intelligence (MAI) Lab, Lagos, Nigeria. Priscu Aloyce Kessy

*Udunna Anazodo*: I am an imaging scientist at the Montreal Neurological Institute in Canada focusing on positron emission tomography (PET) and MRI brain imaging methods development. I’ve been seeking ways to bring these techniques to Africa since my postdoc, and when Johnes gave a presentation at the ISMRM annual meeting in 2019, we got together to figure out how to address the imaging disparity in Africa to improve access, which led to formation of the Consortium for Advancement of MRI Education and Research in Africa (CAMERA) and to many MRI initiatives in Africa. Ugumba is well respected for her expertise in neuroradiology in Tanzania and when she applied to SPARK, I was excited to have her participate and to collaborate with her.

*Johnes Obungoloch*: Udunna is the glue that brings people together. When we met at ISMRM, I was developing portable MRI equipment with a focus on specific applications in Uganda, while Udunna had a broad vision for MRI across Africa. This inspired me to join the network she was trying to build. These networks have enabled us to envision new applications for portable MRI, for example in neuroradiology, which is made possible with collaborations such as with Ugumba.

2. How would you describe the current state of MRI services across Africa?

*Ugumba Kwikima*: There is rising demand for MRI in Africa because of its importance in managing non communicable diseases. There is an increasingly aging population as well as increasing urbanisation, and behavioural trends have been changing, for example in the use of tobacco and alcohol. This has led to ever increasing rates of cardiovascular disease, stroke, epilepsy, dementia and infectious diseases like malaria and HIV which affect the central nervous system. Despite the demand, access to MRI is still poor in Africa. CAMERA conducted a needs assessment survey that showed that there is less than one MRI scanner per million people across Sub-Saharan Africa^2^. Some countries such as the Democratic Republic of Congo and Mali don’t have access to MRI at all. There are many barriers to availability, for example access to stable electricity, and recruiting and retaining skilled personnel to operate the scanners.

*Udunna Anazodo*: We designed the needs assessment survey (NAS) to understand MRI access barriers and how to improve access. This included the educational challenges around training in use of the machines, how they are maintained, the type of services that could be provided and the cost, as well as of course issues with physical access. The COVID-19 pandemic gave us the opportunity to have a high number of respondents from different countries, who had a bit more time during the lockdowns to participate. We also conducted several focused meetings and webinars to gain deeper insights.

**Figure:**
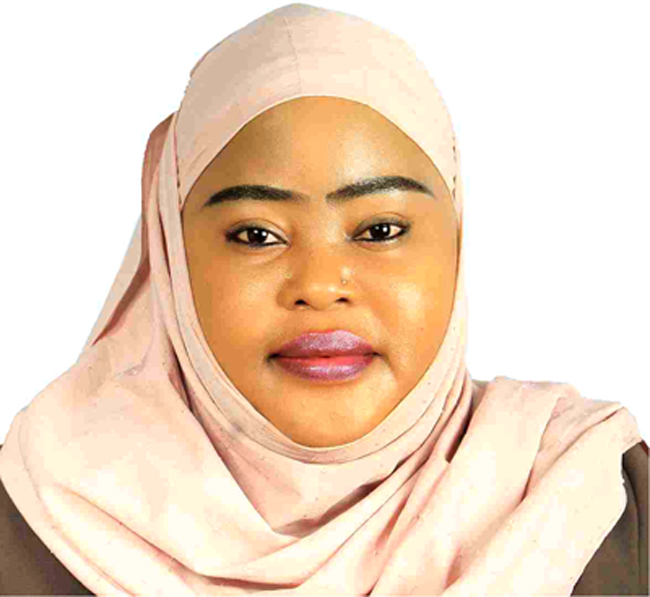
Ugumba Kwikima, Neuroradiologist, Lugalo General Military Hospital, Tanzania. Sandra McPherson

We learnt that overall, Africa is slowly improving access to MRI scanners and has been acquiring a number of 1.5 T MRI field strength systems similar to the clinical systems here in the Global North. This is due to improvements in access to stable electricity and recent availability of several financial schemes to buy scanners. In Kenya, for example, the federal government has a partnership with General Electric (GE) to support finance of scanners which makes them not only more accessible but also more affordable. A similar process is happening in Nigeria where federal government projects are increasing investment in health technologies, including acquisition of MRI scanners. The challenge now is teaching people how to use the scanners well to provide care, especially for the type of problems that are specifically seen in Africa. Training is required to use the machines, but access to sequences and protocols, and knowledge in how to interpret the images is also needed. Many scanners are sold through middlemen, which are often unregulated and sold for inflated prices. I have seen situations where scanners arrive and do not work shortly after. The clinics do not have local technicians or engineers to fix the scanners, so the large amounts of money spent to buy them, sometimes through funds raised through donations, are wasted. The increasing brain drain also makes it challenging to train and retain a critical mass of skilled personnel. This means a specific approach is needed to train and retain imaging experts in Africa.

Furthermore, when it comes to health, the funding model is different in Africa as most people pay out of pocket with limited access to health insurance. This means that patients often borrow money from family members and their community to get an MRI scan that may not be helpful for them - the scan itself may be suboptimal and it may not answer the right questions due to the inadequate imaging environment and lack of skilled experts. It is even more challenging to provide care in this limited system, where patients often present at clinics after the disease has already progressed because of care costs, lack of symptom awareness and stigma, among other reasons. A multifaceted approach is therefore needed to improve imaging access.

*Johnes Obungoloch*: Ugumba and Udunna have answered the questions exhaustively, but I would just add that the services in Africa also focus on structural rather than functional imaging and are concentrated in cities. Another key issue is maintenance, as this is usually subcontracted to people who live outside the country, for example South Africa, Dubai, or Europe. This means service costs can be exorbitant.

**Figure:**
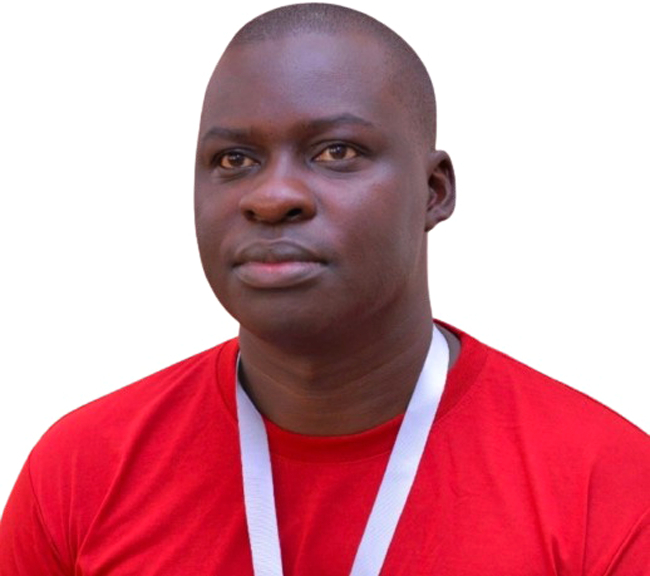
Johnes Obungoloch, Lecturer at Mbarara University of Science and Technology, Uganda. Angella Nakato Muyingo

3. What makes Africa a unique continent for neuroscience and research?

*Ugumba Kwikima:* Most neuroscientific research has been done in Western nations due to access to advanced technologies, and this has led to a significant gap in understanding brain function in non-Western populations, especially Africa. This gap presents an opportunity for fostering culturally responsive neuroscience. As the prevalence of neurological disorders in Africa is increasing, there is a pressing need to increase the quality and quantity of radiological research^3^. There are disparities in the distribution of neuroscience publications, which are coming from a handful of countries such as Tunisia, Morocco, South Africa, and Nigeria. It’s not known what is happening in other countries. In Africa there are several animal models that provide exceptional opportunities to study brain health such as the African green monkey. This model has unique value for genetic and genomic investigations of human disease making it key for studying disease progression, evaluating therapeutic interventions, and developing treatment. This type of research will be able to yield invaluable insights into the understanding and treatment of brain disease.

*Udunna Anazodo:* As Africa has fewer resources than other continents, this provides an opportunity to do neuroscience differently in a more economical, cost-effective manner. It may be possible to design and develop imaging solutions and technologies for resource-constrained settings which can then be adapted later for resource-rich settings – a case of reverse innovation. For example, Johnes is building low-cost and portable brain MRI scanners in Uganda, which will make neuroimaging cheaper and more accessible^4^. This of course brings its own challenges, for example making it low-cost requires the use of lower field strength technology which in turn makes the spatial resolution coarser. But this presents an opportunity to leverage advances in artificial intelligence (AI) to augment portable systems with better image resolution. This technology can then be deployed in the community to answer novel neuroscience questions, for example, to understand the brain health of displaced populations who have left their homelands to flee war, insecurities, and the effects of extreme weather. According to the United Nations Refugee Agency (UNHCR), Africa has the highest proportion of forcibly displaced populations, and most are internally displaced and still seeking refugee within Africa [https://reporting.unhcr.org/global-appeal-2023?page=10]. The majority spend decades displaced and suffer mental health problems that may be helped with accessible diagnostic imaging tools. Africa as a continent provides a unique opportunity to rethink how we do science globally and how to spend research dollars wisely to make maximum impact and be more sustainable. I think Africa provides the opportunity to figure out how to do more neuroscience with less, to increase efficiency.

4. What technological advances do you think could shape the future of MRI provision and positively impact healthcare in Africa?

*Johnes Obungoloch:* I think technological advances must recognise the challenges that there are. As described here and in many publications, this includes low access to energy, highly qualified personnel, and relevant infrastructure such as the Internet. Technology is needed that requires low energy input or consumption and recognises the absence or limited number of scientists and clinicians. AI is one way that may overcome these issues, as well as availability of reliable Internet access and speed which allows contact with individuals outside our countries that are willing to help. Platforms are needed that allow the sharing of information, knowledge, and images with counterparts across borders. Traditional commercial MRI systems have a large physical footprint and weight which requires specialised infrastructure and equipment for their set up - having systems that enable bedside imaging and mobility that allow it to be taken to different communities would also be beneficial. The hope is that this might start in Africa and then come back to the rest of the world.

*Udunna Anazodo:* I agree that AI could be an equaliser if used wisely and responsibly, appreciating the unique challenges^5^. I think it could not only shape the future of MRI imaging but also shape how imaging and MRI are used. One example of this is the integration of AI in MR imaging for rapid disease screening to predict risks for complex conditions such as stroke, dementia, cancer, and other chronic conditions that are now highly prevalent in Africa. Think of how mammograms are routinely used for breast cancer screening or bone densitometry is used to predict fracture risk to introduce interventions early enough to slow down disease progression or minimize the need for expensive treatment such as hip replacement after a fall. In Africa, MRI could be a useful screening tool. If African datasets are included during AI model development for effective integration, that could make MRI a valuable public health tool.

*Ugumba Kwikima:* I think it is worth investing in low field MRI as it is cost effective and doesn’t need a stable power supply. By investing in this approach, AI will also be useful in improving the quality of these images. There are some institutions in Nigeria that have found ways of making power supplies stable and clean, using automated voltage regulators and by maintaining backup.

5. What are some common challenges faced when working to advance MRI provision?

*Udunna Anazodo:* Procurement and access to sequences are common challenges, which make it difficult to translate best practices and standard imaging protocols from the Global North to the Global South. These are often due to porous device regulations that impede manufacturers from directly engaging with and supplying imaging centres.

Then there are challenges in how the devices are used - training and competency in how to use the machines, and specifically how to use them to address their local healthcare needs and within their work environment and situations. Skilled personnel need to be trained and retained to be able to unlock the potential of their scanners to work well to meet their health conditions.

Another issue that faces the entire continent is limited funding support for MRI research. National governments do not adequately fund healthcare research and when they do, they cannot afford to fund expensive healthcare research that involves MRI. Because MRI scanners in Africa are overwhelmingly in clinical practices and reserved mostly for clinical use by clinicians and their staff, there is limited opportunity to conduct research. The clinicians attend to a higher number of patients, and as such do not have enough free time to lead or participate in research. The suboptimal imaging infrastructure and scarcity of MRI scientists, all contribute to the low MRI research in the region. Africa produces roughly 0.5% of global MRI research^2^, although they have one of the largest burdens of disease where MRI is indicated such as cancer, stroke, heart disease, and other neurological conditions.

*Ugumba Kwikima:* Udunna has summarised this well, and I would just add that in Dar es Salaam there are very few scanners which are confined to tertiary hospitals due to limited access to financial capital to support investment in MRI. As there is no quick return, banks are hesitant in giving loans to support health care.

*Johnes Obungoloch:* Another challenge can be a lack of willingness to embrace new technologies. For example, the current practice is to use mid/high field MRI which is available in a few countries, and we are trying to promote low field MRI which is a relatively new technology. Clinical personnel can be quite hesitant and unsure that this technology is worth it, for example by comparing it with images produced with current state of the art 1.5 T or 3 T MRI systems.

6. Can you give an overview of some MRI initiatives that are making a difference?

*Johnes Obungoloch:* The low field MRI systems we are developing have a lot of potential - not as an alternative, but to fill the gap that traditional MRI systems cannot fill in African countries in the foreseeable future. This is an important distinction: low field MRI will be able to augment health care technology provision. There are also initiatives to acquire MRI systems. Most healthcare services should be provided by the government, but the private sector tends to take this over. Still, there are initiatives by different governments to acquire MRI systems. I think an important angle is how you get the technology to the people and how you make people aware of it. That is work that Udunna is doing with CAMERA and the current ISMRM African chapter.

*Udunna Anazodo:* A key part of CAMERA’s framework is advocacy. We are advocating to national governments, funding agencies, device manufacturers, and imaging communities to work together to make scanners more available and join us in training and retaining many local experts. We advocate to funding agencies who have provided long-term support to Africa to fund healthcare delivery, training, and research, to now fund MRI when they provide money to Africa. Most healthcare services provided by the government rely on funding from outside Africa to run, and these usually gets diverted to maternal and childcare, and infectious disease. But cancer, stroke, heart disease are at the bottom of the priority list. We have recently started a podcast [https://www.cameramriafrica.org/spotlight] to promote MR research and practice in Africa and give clinicians and researchers an opportunity to highlight their work.

CAMERA’s recent Scan with Me (SWiM) training program is another example of industry partnerships to upskill imaging technologists on best practices for image acquisition, including protocol optimization. Siemens Healthineers, Erlangen, Germany and Canon Medical Systems, USA provided free hands-on demonstrations to SWiM participants on how to acquire high quality images on their scanners, to close gaps in scanner application training. Thanks to this valuable vendor support, the SWiM technologists were able to adapt existing MRI protocols on their scanners and tailor them to produce high quality images at significantly shorter scan times (50% reduction in some cases), improving their clinical workflow and ability to provide care to more people. These scan protocols are available for anyone, anywhere in the world to freely use, particularly those in low-resource settings [https://github.com/CAMERA-MRI/SWiM]. SWiM and other CAMERA training initiatives, such as SPARK, are not only linking device manufacturers and imaging software providers directly with health centres in Africa, but they are also more importantly connecting centres together and with basic scientists within countries and across the continent, to enable a collaborative environment for MRI to thrive. With this type of strong communication and collaboration, policies, and training solutions are feasible to advance MRI in Africa. For example, Dr Kwikima participated in SPARK and learned alongside radiology, neurosurgery, and oncology residents, as well physics and computer science students from 12 African countries on how to build AI models for brain tumour segmentation on MRI. The network established among disciplines and countries has spurred collaborative research including curation of larger brain MRI data for future AI applications and development of a recommended guideline for brain tumour imaging in Africa, which Dr Kwikima is leading.

Last year, the Own Your Future Mentorship (OYFM) program at CAMERA was established, to empower emerging clinicians and researchers to see a stable, steady career in MRI in Africa, which could help retain talent. OFYM is focused on radiology residents and fellows, and physicists and biomedical engineers interested in MRI. It taps into the network that CAMERA built including MRI experts across the world to help mentor and train a large pool of mentees. The program uses monthly seminar series and regular one-on-one mentoring to transfer clinical, physics (including research), and entrepreneurship skills. The goal is to guide early career clinicians and researchers to build and sustain successful careers within Africa, supported by the network of fellow mentees across the continent and imaging experts from around the world. Up to 80% of attendees to the seminar series reported improvement in their knowledge and skills on the topic. The one-on-one mentoring is set to begin this Spring, and a pilot run of the OYFM focused mentoring approach was successful completed and resulted in a recent publication on brain imaging between OYFM Mentee, Dr Afolabi Ogunyele, a radiology resident at a Nigerian teaching hospital, and OYFM Mentor, Prof. Vera Keil, a renowned Neuroradiologist from Amsterdam University Medical Center, The Netherlands^6^.

These initiatives will cumulatively improve MRI access in Africa, especially through retention of local expertise. People often leave Africa because they have been trained but can’t apply their knowledge because of problems with infrastructure. They go elsewhere where they can use their knowledge, typically in the Global North. CAMERA is committed to continue to train and mentor a critical mass of people in Africa who will be skilled to train others and form intra- and cross-country networks to sustain valuable MRI care in the region.

*Ugumba Kwikima:* Participating in the SPARK Academy really opened my eyes as a clinician. I gained a lot of knowledge regarding artificial intelligence in the diagnosis and management of brain tumours, and reading about AI now after gaining this knowledge has enabled me to brainstorm about new directions.

7. What is your vision for MRI in Africa a decade from now?

*Johnes Obungoloch:* I think there will be a marked improvement in access to MRI for most of the population in Africa a decade from now, also in terms of research and collaboration. I think there is good momentum right now, and if there is still this momentum a decade from now, there could be hundreds of researchers across Africa with expertise in MRI research and clinical work. In terms of technology itself, I hope to see at least some manufacturing of MR technology locally. If not the full technology itself, then at least the components. This will come along with expertise in maintenance, building and repair of these services. This would also open opportunities for businesses and further training. I also hope there will be development of standards and regulations that are more considerate of the African continent, for instance of the situations faced locally. If these standards were developed locally, they may make promotion of the technology easier in the future.

*Udunna Anazodo:* I believe that a decade from now MRI will be largely driven by reverse innovation, to again borrow a term I learnt from Dr Jha (Harry) Saurabh, a radiologist at the University of Pennsylvania. This refers to the idea that innovation implemented to meet the needs of low-income countries can then be adapted in high-income countries to improve sustainability. I think Africa will lead this new approach to innovation in MRI, not only in low field methods but also low-cost AI solutions. AI’s potential could be unlocked when it is used in the lowest resource settings. A decade from now there will also be a lot of MRI research coming out of Africa with many novel applications including routine use in infectious disease and in public health to inform primary and secondary prevention practices. I hope this will all be made possible with a pool of talent and a good relationship between local health centres, industry partners, government policy agencies and publishing bodies, and the global MRI community.

This interview was conducted by Dr. Henrietta Howells.
